# QTL Mapping of Seed Quality Traits in Crops

**DOI:** 10.3390/plants14030482

**Published:** 2025-02-06

**Authors:** Moulay Abdelmajid Kassem

**Affiliations:** Plant Genomics and Biotechnology Laboratory, Department of Biological and Forensic Sciences, Fayetteville State University, Fayetteville, NC 28301, USA; mkassem@uncfsu.edu

The ability to map quantitative trait loci (QTLs) has revolutionized plant genetics, providing an essential toolkit for dissecting the genetic basis of agronomic traits. This Special Issue, “QTL Mapping of Seed Quality Traits in Crops”, published in the journal Plants, features 11 cutting-edge research articles that exemplify the current advances in QTL mapping and its application to seed quality traits, including protein content, sugar accumulation, frost tolerance, seed longevity, and more. These contributions highlight the importance of QTL mapping as a powerful approach to improve seed quality, which is crucial for food security, nutritional improvement, and environmental adaptability in crops.

## 1. Advances in Understanding Seed Quality Traits Across Crop Species

Seed quality traits are often complex, influenced by multiple genetic loci and environmental factors. This complexity necessitates robust tools and methodologies, such as QTL mapping and candidate gene identification, to unravel the genetic architecture underlying these traits. The articles in this Special Issue reflect a diverse set of approaches and crop species, from soybean and wheat to common bean and mustard. Together, they provide an invaluable resource for researchers and breeders striving to improve seed quality.

## 2. Highlights of the Contributions

### 2.1. Protein and Nutritional Composition in Soybean

Soybean, a major global source of plant-based protein, has been the focus of multiple studies in this issue. Two recombinant inbred line (RIL) populations were studied in relation to seed protein content [1], revealing QTLs and candidate genes associated with this critical trait. Additionally, QTL mapping for seed sugar contents in the well-studied soybean population ‘Forrest’ × ‘Williams 82’ identified genomic regions influencing seed carbohydrates [2].

### 2.2. Frost Tolerance and Seed Longevity in Wheat and Capsicum

Understanding environmental stress responses is critical for sustainable agriculture. The identification of novel QTLs associated with frost tolerance in winter wheat [3] and seed longevity in *Capsicum annuum* [4] provides valuable insights into breeding crops resilient to cold climates and storage conditions.

### 2.3. Seed Morphology and Biochemical Composition

Digital image processing techniques were applied to assess seed size, shape, and color in bread wheat, enabling precise phenotypic measurements and QTL mapping [5]. Similarly, tocopherol content in soybean [6] and glucosinolate accumulation in mustard [7] were mapped to candidate loci, advancing our understanding of seed biochemical traits.

### 2.4. Diversity in Common Bean and Wheat

Natural variation in Portuguese common bean populations provided insights into the genetic architecture of nutritional traits [8], while high-density SNP-based genetic linkage maps were utilized to identify QTLs for grain traits in wheat [9].

### 2.5. Integration of Databases and Bioinformatics

Modern plant breeding relies heavily on data integration and bioinformatics. An article focused on leveraging crop databases for candidate gene identification [10] underscores the increasing role of computational tools in QTL analysis.

### 2.6. Isoflavone and Nutraceutical Properties in Soybean

Isoflavones are bioactive compounds with significant health benefits. Mapping QTLs for seed isoflavone content in soybean [11] highlights the potential for enhancing nutraceutical properties through targeted breeding.

## 3. Looking Ahead: Bridging the Gap Between Research and Application

The findings presented in this Special Issue demonstrate how QTL mapping has matured as a discipline, integrating high-throughput phenotyping, genomics, and bioinformatics. While significant progress has been made, the transition from mapping to application in breeding programs remains a challenge. Bridging this gap will require greater emphasis on candidate gene validation, functional genomics, and the incorporation of genomic selection in breeding pipelines.

## 4. Concluding Remarks

As the global population grows and climate change exerts increasing pressure on agricultural systems, the need for high-quality seeds is becoming ever more critical. The research presented in this Special Issue represents a step forward in addressing this challenge, offering novel genetic insights and practical tools for breeding programs. I hope these studies will inspire further research and innovation in QTL mapping and beyond.

## References

[1] Park H.R., Seo J.H., Kang B.K., Kim J.H., Heo S.V., Choi M.S., Ko J.Y., Kim C.S. (2023). QTLs and Candidate Genes for Seed Protein Content in Two Recombinant Inbred Line Populations of Soybean. Plants.

[2] Knizia D., Bellaloui N., Yuan J., Lakhssasi N., Anil E., Vuong T., Embaby M., Nguyen H.T., Mengistu A., Meksem K. (2023). Quantitative Trait Loci and Candidate Genes That Control Seed Sugars Contents in the Soybean ‘Forrest’ by ‘Williams 82’ Recombinant Inbred Line Population. Plants.

[3] Bolouri P., Haliloğlu K., Mohammadi S.A., Türkoğlu A., İlhan E., Niedbała G., Szulc P., Niazian M. (2023). Identification of Novel QTLs Associated with Frost Tolerance in Winter Wheat (*Triticum aestivum* L.). Plants.

[4] Arif M.A.R., Tripodi P., Waheed M.Q., Afzal I., Pistrick S., Schütze G., Börner A. (2023). Genetic Analyses of Seed Longevity in *Capsicum annuum* L. in Cold Storage Conditions. Plants.

[5] Arif M.A.R., Komyshev E.G., Genaev M.A., Koval V.S., Shmakov N.A., Börner A., Afonnikov D.A. (2022). QTL Analysis for Bread Wheat Seed Size, Shape and Color Characteristics Estimated by Digital Image Processing. Plants.

[6] Knizia D., Yuan J., Lakhssassi N., El Baze A., Cullen M., Vuong T., Mazouz H., Nguyen H.T., Kassem M.A., Meksem K. (2022). QTL and Candidate Genes for Seed Tocopherol Content in ‘Forrest’ by ‘Williams 82’ Recombinant Inbred Line (RIL) Population of Soybean. Plants.

[7] Tandayu E., Borpatragohain P., Mauleon R., Kretzschmar T. (2022). Genome-Wide Association Reveals Trait Loci for Seed Glucosinolate Accumulation in Indian Mustard (*Brassica juncea* L.). Plants.

[8] Mendes F.A., Leitão S.T., Correia V., Mecha E., Rubiales D., Bronze M.R., Vaz Patto M.C. (2022). Portuguese Common Bean Natural Variation Helps to Clarify the Genetic Architecture of the Legume’s Nutritional Composition and Protein Quality. Plants.

[9] Lv D., Zhang C., Yv R., Yao J., Wu J., Song X., Jian J., Song P., Zhang Z., Han D. (2021). Utilization of a Wheat50K SNP Microarray-Derived High-Density Genetic Map for QTL Mapping of Plant Height and Grain Traits in Wheat. Plants.

[10] Brown A.V., Grant D., Nelson R.T. (2021). Using Crop Databases to Explore Phenotypes: From QTL to Candidate Genes. Plants.

[11] Knizia D., Yuan J., Bellaloui N., Vuong T., Usovsky M., Song Q., Betts F., Register T., Williams E., Lakhssassi N. (2021). The Soybean High Density ‘Forrest’ by ‘Williams 82’ SNP-Based Genetic Linkage Map Identifies QTL and Candidate Genes for Seed Isoflavone Content. Plants.

